# Fabry disease in the Spanish population: observational study with detection of 77 patients

**DOI:** 10.1186/s13023-018-0792-8

**Published:** 2018-04-10

**Authors:** Irene Vieitez, Olga Souto-Rodriguez, Lorena Fernandez-Mosquera, Beatriz San Millan, Susana Teijeira, Julian Fernandez-Martin, Felisa Martinez-Sanchez, Luis Jose Aldamiz-Echevarria, Monica Lopez-Rodriguez, Carmen Navarro, Saida Ortolano

**Affiliations:** 1 Rare Diseases and Pediatric Medicine Research Group, Galicia Sur Health Research Institute (IIS Galicia Sur). SERGAS-UVIGO, Vigo, Spain; 2 Institute of Cellular Biology, University Medical Center Goettingen, Goettingen, Germany; 3 Department of Pathology, Xerencia de Xestion Integrada de Vigo, Sergas, Vigo, Spain; 4 Department of Internal Medicine, Xerencia de Xestion Integrada de Vigo, Sergas, Vigo, Spain; 5 Department of Nephrology, Hospital of Torrecardenas, Almeria, Spain; 6 Metabolic Unit, Hospital de Cruces, Barakaldo, Spain; 7 Coordinadora Grupo Trabajo de Enfermedades Minoritarias de la SEMI, Madrid, Spain; 8 Department of Pathology, Institute of Biomedical Research of Vigo, Clinical Emeritus, University Hospital of Vigo, Vigo, Spain; 9 Galicia Sur Health Research Institute (IIS Galicia Sur) Hospital Alvaro Cunqueiro, Pl.2 Bloque tecnico, zona A, Estrada Clara Campoamor 341, 36312 Vigo, Pontevedra Spain

**Keywords:** Fabry disease, Lysosomal storage disorders, Enzymatic screening, Intronic variants, *GLA* complex haplotype

## Abstract

**Background:**

Fabry disease is a multisystemic lysosomal storage disorder caused by the impairment of α-galactosidase A. The incidence of this rare disease is underestimated due to delayed diagnosis. Moreover, the management of the identified subjects is often complicated by the detection of variants of unclear diagnostic interpretation, usually identified in screening studies. We performed an observational study based on biochemical and genetic analysis of 805 dried blood spot samples from patients with clinical symptoms or family history of this pathology, which were collected from 109 Spanish hospitals, all over the country.

**Results:**

We identified 77 new diagnosed patients with mutations related to classical Fabry disease, as well as 2 subjects with c.374A > T; p.His125Leu, a possible new mutation that need to be confirmed. Additionally, we detected 8 subjects carrying genetic variants possibly linked to late onset Fabry disease (p.Arg118Cys and p.Ala143Thr), 4 cases with polymorphism p.Asp313Tyr and 36 individuals with single nucleotide polymorphisms in intronic regions of *GLA*. Five of the identified mutations (c.431delG; c.1182delA; c.374A > T; c.932 T > C; c.125 T > A; c.778G > A), which were associated with a classical phenotype have not been previously described. Moreover 3 subjects presenting complex haplotypes made up by the association of intronic variants presented impaired levels of *GLA* transcripts and Gb3 deposits in skin biopsy.

**Conclusions:**

Enzymatic screening for Fabry Disease in risk population (2 or more clinical manifestations or family history of the disease) helped to identify undiagnosed patients and unravel the impairment of *GLA* expression in some subjects with complex haplotypes.

**Electronic supplementary material:**

The online version of this article (10.1186/s13023-018-0792-8) contains supplementary material, which is available to authorized users.

## Background

Fabry disease (FD, OMIM#301500) is an X-linked lysosomal storage disorder (LSD), caused by the impairment of α-galactosidase A (α-Gal A, EC 3.2.1.22), which leads to the progressive accumulation of globotriaosylceramide (Gb3), and its derivatives (eg. lyso-Gb3). To date, over 700 genetic defects have been identified in *GLA,* however no frequent mutations were described and it is difficult to establish a genotype-phenotype correlation [1]. The classical form of FD is generally associated with a very low residual α-Gal A activity in men (< 1%, ~ 1 μmol/Lh). Glycosphingolipids are mainly accumulated in the vascular endothelium and other cells of skin, autonomic nervous system, kidney, brain and gastrointestinal tract, among others. Electron microscopy examination of skin biopsy has proved to be a significant diagnostic tool in FD, since deposits of Gb3 are morphologically characteristic and accumulate in several type of dermal cells, reflecting the deposits in vital organs such as heart or kidney [2, 3].

Common symptoms and signs of FD include acroparesthesias, angiokeratomas, hypohidrosis, *cornea verticillata*, chronic renal failure, left ventricular hypertrophy, hypertrophic cardiomyopathy, arrhythmia and stroke. Nevertheless, some *GLA* variants such as p.Ala143Thr and p.Arg118Cys, have been related to late onset FD, which is characterized by a higher α-Gal A residual activity (> 1%) and exclusively cardiac or renal manifestations [4, 5]. Due to a selection bias in the screening studies, these variants are of unclear diagnostic interpretation which complicates the management of the identified subjects [6] and precluded up to date the inclusion of FD in the uniform screening panels recommended by the Advisory Committee on Hereditable Disorders in Newborns and Children [7].

Moreover, the poor specificity of FD phenotype often results in delayed diagnosis, which is usually established in adulthood, even if the symptoms started during childhood [8]. It was originally reported that the incidence of FD is of 1:117,000 [9] for the general population; however, pilot studies performed in asymptomatic newborns estimated an incidence ranging between 1:3000 and 1:7000 live births [10, 11]. These data and the results of several screening studies performed in patients with medical conditions of diverse etiology (i.e. stroke, renal failure, ventricular hypertrophy) [4, 12, 13] highlight the large number of undetected FD patients, who are probably still unidentified or misdiagnosed.

Avoiding delayed diagnosis is crucial to increase the quality and expectancy of life of the patients who need a specific treatment. In Europe there are, currently, three available treatments with specific indication for FD (agalsidase alfa, agalsidase beta and migalastat hydrochloride). These drugs are more efficient in improving patients’ condition when the therapy starts before extensive tissue degeneration is achieved [14, 15].

The aim of this work was to perform an observational study based on biochemical and genetic analysis of subjects who manifest a combination of symptoms and signs suggestive of FD (i.e. acroparesthesia, cardiac hypertrophy, stroke in the young, renal failure) or have a family history of the disease. This will allow the identification of FD patients with non-specific diagnosis, who can therefore access more appropriate therapeutic protocols. The results of the study will also allow to define appropriate criteria and parameters to design a future validated diagnostic study.

## Methods

### Design of the study

We carried out an observational study based on biochemical screening of α-Gal A and *GLA* sequence in subjects with age comprised between 1 and 85 years (average age 47.05 ± 18.53, standard deviation (SD)), who presented more than one clinical symptom or sign associated to FD (i.e. ventricular hypertrophy, renal insufficiency, stroke in the young, acroparesthesia, angiokeratome, intolerance to heath or cold, neuropathic pain, hypertrophic myocardiopathy), or who were genetically-linked relatives of previously diagnosed patients. Enzyme activity was quantified in dried blood spots samples (DBS) by fluorimetric assay. A confirmatory genetic test was performed in male subjects with activity < 2.6 μmol/Lh and in all the females. The cut off value for enzymatic activity in male subjects was estimated comparing α-Gal A activity data obtained in 5 males with definitive diagnosis of FD and 15 healthy volunteers. Threshold was established considering percentile of positive patients and adding 0.5 to increase the sensitivity of the assay.

### Patients

We collected 805 dried blood spot samples (479 males and 326 females) coming from different clinical departments of 109 Spanish hospitals along a period of 6 years (2009–2014) (Additional file 1: Figure S1). For each patient, relevant clinical symptoms and signs were listed by the responsible physician by checking boxes in the sample collecting cards especially designed for this study (Additional file 1: Figure S2). All patients (or their legal tutors) signed the informed consent and the study was approved by the Galician Clinical Research Ethics Committee (Ref 2009/182, up-date on March 29th 2011).

### Enzyme activity assay

α-Gal A activity was measured following the method of Chamoles et al. [16]. Briefly, a fluorimetric assay was performed on triplicates DBS fragments of 2 mm diameter in buffer Citrate-Phosphate 0.15 M pH 4.2, using 4-methylumbelliferyl-galactopiranoside (2.5 mM) as a substrate and in presence of N-acetylgalactosamine (0.25 M). Enzymatic activity was calculated referring to a standard curve of 4-methylumbelliferon and expressed as μmol of substrate per hour and liter of blood (μmol/Lh) ± SD.

### Genetic sequencing

The seven exons of the *GLA* gene were amplified by PCR with specific primers (Additional file 1: Table S1) and sequenced by Sanger method on an AB Prism 310 Genetic Analyzer (Applied Biosystems). Results were analyzed using the software Chromas 2.4. The new mutations have been analyzed with an exhaustive in silico analysis using combined computer algorithms to predict their potential pathogenic status (Polyphen-2, Sort Intolerant from Tolerant, Pmut and MutationTaster2 software [17–20]). We classified the new variants as pathogenic mutations when they are considered as disease causing by at least three software tools and they meet criteria for definitive diagnosis (high Gb3-LysoGb3 levels, presence of lamellar bodies or are males with low α-Gal A activity).

### Biopsy

A 6 mm punch skin biopsy was obtained from an unaffected sub-axillary area and analyzed by immunohistochemistry, as previously described [2]. This analysis was performed, to explore if Gb3 deposits were formed, in two of the patients of the cohort, who had reduced α-Gal A activity and a complex intronic variant in *GLA.* Cryocutted sections of 7 μm were fixed in PFA 4% for 2 h and permeabilized with Triton 0.1% *v*/v diluted in phosphate buffer saline with bovine serum albumin 5% p/v. Slides were incubated over night at 4 °C with primary antibody Anti-human CD77 (BD Pharmingen#551352) and after rinse they were incubated with FITC goat anti mouse IgG/IgM (#555988, BD Pharmingen) for 2 h at room temperature. Phalloidin-Rodhamine (Sigma #94072) and DAPI (Sigma #D8417) were used to stain respectively cellular membranes and nuclei. Samples were examined with a fluorescence microscope (LeicaDMI 6000B).

### Real time PCR

Leukocytes from three patients with reduced α-Gal A activity and an association of intronic SNPs in *GLA* were obtained to measure gene transcript levels. RNA was extracted with an RNAeasy kit (QIAGEN) and retrotranscribed with Omniscript kit (QIAGEN) and an oligodT primer. mRNA levels were detected by q-PCR using Taqman probe *GLA* #Hs04183387. Transcript amounts were calculated with reference to the housekeeping gene GAPDH #Hs02758991, using the method of the amplification cycles (ΔΔCt), described by Pfaffl [21] and normalizing values to the ones obtained for the calibration sample (value of 1), (mRNA extracted from a healthy volunteer). Three independent experiments were repeated for each of the subjects using calibration samples from 2 different control individuals.

### Western blot

Blood samples from two patients with reduced α-Gal A activity and complex intronic haplotype in *GLA* were obtained to analyze α-Gal A protein expression levels*.* Leukocytes were purified by peripheral blood with Ficoll and lysed with RIPA buffer containing protease inhibitor cocktail (# P1860, Sigma-Aldrich). Following sonication and centrifugation, the supernatant was diluted with Laemmli buffer and loaded on 12% Tris-Glycine-Acrylamide gels for SDS-PAGE. Proteins were transferred to PVDF membranes and blotted with anti-human-ɑ-GalactosidaseA antibody (#ab129173, Abcam). Membranes were treated with HRP-conjugated secondary antibody (#NA934V, GE) and revealed with ECL plus (#RPN2232, GE). Images were taken with an IMAGE QUANT 350, GE analyzer and α-Gal A expression was qualitatively compared with actin expression in each sample.

### Analysis

Positive predictive value, method accuracy and confidence interval for the enzymatic assay, in the male cohort, were calculated using EpiDat 3.1 (Xunta de Galicia)**.** We considered as true positive exclusively the subjects with validated diagnosis (Table 1). Categorical variables are expressed as mean ± SD. *GLA* transcript levels were analyzed by ANOVA test followed by student t-test to compare groups of mean ± standard deviation. Statistical significance was assumed at *p* < 0.05. Gb3 deposits were quantified using Leica LAS v.2.0, morphometric software by describing and counting regions of interest.

**Table 1:** Patients with definitive diagnosis of Fabry Disease. Age at diagnosis is indicated. M: hemizygous men F: heterozygous females. Activity of ɑ-Gal A is expressed in μmol/Lh. FS: family study; X: not indicated, NM. not measured; Symptoms 1: Renal Failure 2: Ventricular Hypertrophy; 3: Acroparesthesia, 4: Angiokeratome; 5: Cardiomegaly; 6:*Fabry facies*; 7: Hernia; 8: Hypohidrosis; 9: Intolerance to heat or cold; 10: Neuropathic pain; 11: Corneal clouding; 12: Stroke in the young, 13: Hypertrophic, myocardiopathy

*Code*	*Age*	*Sex*	*Medical Department*	Symptoms	*ɑ-Gal A Activity*	*Mutation*	Reference
1	52	M	Nephrology	1, 2,13	0.59 ± 0.14	c.613C > T; p.Pro205Ser	[35]
2	30	M		FS	0.76 ± 0.43		
3	46	F		FS	2.97 ± 0.16		
4	25	M		FS	1.27 ± 0.06		
5	56	F		FS	3.33 ± 0.33		
6	47	F		FS	4.36 ± 1.58		
7	76	F		FS	1.83 ± 0.24		
8	55	F		FS	2.24 ± 0.02		
9	24	F		FS	1.82 ± 0.38		
10	22	F		FS	0.58 ± 0.37		
11	29	M		FS	1.04 ± 0.07		
12	62	F		FS	4.40 ± 0.03		
13	44	F		FS	3.67 ± 1.45		
14	60	M		FS	0.28 ± 0.11		
15	82	F		FS	6.41 ± 0.01		
16	21	M		FS	1.04 ± 0.61		
17	34	M		FS	0.48 ± 0.28		
18	0.5	F		FS	3.7 ± 1.73		
19	8	M	Genetics	FS	0.92 ± 0.1	c.713G > A; p.Ser238Asn	[4]
20	12	M		FS	1.2 ± 1.81		
21	57	M		FS	1.12 ± 0.12		
22	X	F		FS	3.76 ± 0.36		
23	52	F		FS	2.47 ± 0.22		
24	31	F		FS	2.54 ± 0.05		
25	39	F	Pediatrics	FS	4.98		
26	2	F		FS	1.30		
27	4	M		FS	0.69		
28	74	M	Cardiology	2, FS	0.58 ± 0.38		
29	49	F		2,3	1.01 ± 0.34		
30	52	M		2, FS	0.56 ± 0.25		
31	15	F		FS	4.68 ± 0.30		
32	X	F		FS	4.61 ± 0.42		
33	10	F	Nephrology	FS	4.75 ± 0.11		
34	39	M		FS	1.35 ± 0.01		
35	70	M		FS	1.48 ± 0.56		
36	64	M		FS	1.89 ± 0.65		
37	21	M		FS	1.45 ± 0.7		
38	17	F		FS	NM		
39	X	M		FS	1.67 ± 0.12		
40	38	M		FS	1.20 ± 0.10		
41	56	F		FS	2.4 ± 0.07		
42	62	F		FS	4.9 ± 1.16		
43	30	M	Internal Medicine	2,4,6,10	1.27 ± 0.10	c.836A > G; p.Gln279Arg	[36]
44	28	M		2,10	0.92 ± 0.2		
45	51	F		2,4,6,10	4.02 ± 0.01		
46	26	M		FS	1.5 ± 0.74		
47	53	M		2	1.98 ± 0.44		
48	48	F		FS	3.13 ± 0.32		
49	48	F		2	7.45 ± 0.36		
50	23	F		FS	7.13 ± 0.56		
51	22	M	Internal Medicine	3,4,5,6,7,8,9,10	0.75 ± 0.05	c.422C > T; p.Thr141Ile	[37]
52	55	F		2,4,5	1.01 ± 0.01		
53	53	M		3,4,5,9,10	2.25 ± 0.06		
54	48	M	Cardiology	2	1.05 ± 0.54	c.679C > T; p.Arg227*	[38]
55	48	M		1,2	0.86 ± 0.19		
56	46	M	Cardiology	2	1.19 ± 0.05	c.242G > C; p.Trp81Ser	[36]
57	13	F	Internal Medicine	3	1.48 ± 0.3	c.242G > C; p.Trp81Ser	[36]
58	12	F	Nephrology	FS	3.16 ± 0.2	c.1232G > A; p.Gly411Asp	[39]
59	65	F		FS	5.95 ± 1.25		
60	27	M	Nephrology	1,6,10	0.94 ± 0.3	c.778G > A; p.Gly260Arg	Not described
61	59	F		12	3.58 ± 0.59		
62	53	M	Neurology	1,5	0.13 ± 0.01	c.374A > T; p.His125Leu	Not described (possible new mutation that need to be confirmed)
63	77	F		FS	1.04 ± 0.42		
64	X	M	Genetics	FS	1.9 ± 0.84	c.463G > C; p.Asp155His	[40]
65	X	F		FS	NM		
66	42	M	Cardiology	FS	1.87 ± 0.53	p.Arg227Gln	[41]
67	X	M		FS	1.96 ± 0.47		
68	35	F	Internal Medicine	1,3,4,9	5.88 ± 0.37	c.431delG; p.Gly144Alafs*21	Not described
69	48	M	Genetics	1,10,13	0.84 ± 0.18	c.1182delA; p.Phe396Serfs*8	Not described
70	37	M	Nephrology	1	0.63 ± 0.03	c.509A > T; p.Asp170Val	[42]
71	23	F	Internal Medicine	3,8,11	1.02 ± 0.13	c.1277_1278delAA; p.Lys426Argfs*11	[43]
72	19	F	Internal Medicine	FS	2.43 ± 0.58	c.132G > T; p.Trp44Cys	[44]
73	X	M	Internal Medicine	2,3,4,8,9,10	1.21 ± 0.54	c.932 T > C; p.Leu311Pro	Not described
74	35	F	Nephrology	3,6,10,11	1.35 ± 0.01	c.155G > C; p.Cys52Ser	[45]
75	46	M	Internal Medicine	2,4,8	0.85 ± 0.34	c.572 T > C; p.Leu191Pro	[46]
76	71	F	Genetics	1,10	NM	c.337 T > C; p.Phe113Leu	[42]
77	43	F	Nephrology	FS	8.37 ± 0.84	c.298A > T; p.Arg100*	[47]
78	55	F	Cardiology	9,4	7.61 ± 0.63	c.53 T > G; p.Phe18Cys	[48]
79	27	M	Clinical Pathology	1, 12	1.98 ± 0.25	c.125 T > A; p.Met42Lys	Not described

## Results

Enzymatic activity was measured in 805 DBS samples of 479 males and 326 females; among them, *GLA* sequencing was performed in male patients with activity lower than 2.6 μmol/Lh and in all female subjects. We detected 77 patients with a genetically confirmed diagnosis of FD (37 males and 40 females) belonging to 21 different families. Five of the detected mutations that we associated to FD had not been previously described at the time of diagnosis (c.431delG; c.1182delA; c.374A > T; c.932 T > C; c.125 T > A; c.778G > A). We also identified a family of 2 subjects (1 male and 1 female), who presented with clinical features compatible with the classic form of FD, and carry a newly described variant in *GLA* (c.374A > T;p.His125Leu), which need to be further studied to confirm its pathogenic status. More details about these subjects are described in Table 1.

The c.431delG frameshift mutation was identified in a subject belonging to a family of FD patients, who had been diagnosed by enzyme assay. The clinical features of this family (eg. angiokeratomas, acroparestesias, cardiac hypertrophy and renal dysfunction) were described by Barba-Romero et al. [22]. Ultrastructural analysis of skin biopsies from two previously identified members of the family revealed the presence of abundant zebra bodies in different structures (fibroblast, blood vessel, smooth muscle and nerve endings) [3].

The c.1182delA was a de novo mutation found in a 47-year-old man (Patient #69), presenting multiple parapelvic cysts in both kidneys, serum creatinine of 1.6 mg/dl and proteinuria of 1 g/24 h, who eventually underwent renal failure and transplant. The patient also presented with cardiomyopathy, small fiber neuropathy, *cornea verticillata* and hypohidrosis. Low levels of α-Gal A activity were confirmed, following measurement in plasma (0.5 nmol/h/ml) and leukocytes (4.2 nmol/h/mg), so enzyme replacement therapy was started [23]. This frameshift mutation will causes the substitution of 6 amino acids in the C-terminal part of the protein and the formation of a premature stop codon (26 amino acids are missing compared to the wild-type sequence) (Additional file 1: Figure S3). As for the majority of the frameshift mutations, the transcript carrying this variant is most likely degraded at the endoplasmic reticulum level due to instability.

Patient #73 with the c.932 T > C; p.Leu311Pro presented several clinical manifestations related to classical FD, such as ventricular hypertrophy, acroparesthesias, angiokeratoma, hypohidrosis, neuropathic pain with intolerance to heat or cold and renal impairment (albuminuria 160 μg/min). α-Gal A activity was also measured in plasma (1.1 nmol/ml/h) and leukocytes (0.13 nmol/mg/h), while Gb3/Ga2 ratio was 1.6. Since 2011 this patient is under enzyme replacement therapy.

Patient #79 with the c.125 T > A; p.Met42Lys mutations presented mainly neurological symptoms (acute ischemic stroke with lacunar hemibulbar lesion) associated to chronic renal dysfunction with increased proteinuria/creatinine ratio (1.50 mg/mg). The patient also present with classic hallmarks of FD, such as angiokeratomas and *cornea verticillat*a. The diagnosis was confirmed by measuring Lyso-Gb3 levels in plasma, which were quantified in 16 ng/ml (Cut-off values 0–3.5).

Patient #60 with the c.778G > A; p.Gly260Arg presented severe peripheral sensorial neuropathy, hypohidrosis, *cornea verticillata,* microalbumin, abdominal pain and multiple inflammatory mesenteric and inguinal adenopathies confirmed by biopsy, which also showed evidences of cellular deposits. This patient is under enzyme replacement therapy since 2011 [24]. Patient #61, was identified by familial study, following definitive diagnosis of the index case [#60] in his center of origin.

The subject #62, who bears the c.374A > T; p.His125Leu mutation, was derived from a neurology department and presented with stroke at young age associated to renal insufficiency and cardiomegaly, this patient underwent Enzyme replacement therapy. His mother (subject #63) is heterozygous for the same mutation. ⁠

Additional 51 cases (6 males and 45 females) presented genetic variants in *GLA* of uncertain significance*.* Polymorphism p.Asp313Tyr (c.937G > T) was found in 7 samples (4 males and 3 females). We also detected the controverted late-onset variants p.Ala143Thr (c.427G > A, found in 3 females and 1 male) and p.Arg118Cys (c.352C > T, found in 4 females) (Table 2).

**Table 2:** Subjects with late onset variants in *GLA* or p.Asp313Tyr polymorphism, Activity of ɑ-Gal A is expressed in μmol/Lh ± Standard deviation. Age at diagnosis is indicated. M: hemizygous men F: heterozygous females. FS: family study; X: not indicated, NM. Not measured; Symptoms 1: Renal Failure 2: Ventricular Hypertrophy; 3: Acroparesthesia, 4: Angiokeratome; 5: Cardiomegaly; 6: *Fabry Facies*; 7: Hernia; 8: Hypohidrosis; 9: Intolerance to heat or cold; 10: Neuropathic pain; 11: Corneal clouding; 12: Stroke in the young, 13: Hypertrophic, myocardiopathy

*Code#*	*Age*	*Sex*	*Medical Department*	Symptoms	*ɑ-Gal A Activity*	*Variant*
80	42	F	Neurology	FS	3.6 ± 1.76	c.352C > T;p.Arg118Cys
81	51	F		FS	2.33 ± 0.43	
82	28	F		FS	5.83 ± 0.13	
83	31	F		FS	6.64 ± 0.13	
84	X	M	Genetics	6,13	4.21 ± 2.02	c.427G > A; p.Ala143Thr
85	45	F		FS	NM	
86	X	F		2,13	NM	
87	17	F	Neurology	FS	3.05 ± 0.17	
88	75	M	Cardiology	FS	3.77 ± 0.22	c.937G > T; p.Asp313Tyr
89	57	F	Nephrology	FS	4.28 ± 0.79	
90	X	M	Genetics	FS	5.2 ± 0.87	
91	35	M	Neurology	FS	3.8 ± 1.57	
92	1	F	Nephrology	1	8.69 ± 1.93	
93	48	M	Neurology	3,5	5.5 ± 0.41	
94	45	F	Clinical Pathology	2,5	6.63 ± 0.56	

Moreover, single nucleotide polymorphisms (SNPs) in intronic sequences were detected in 36 individuals (1 male and 35 females). Complex haplotypes made up of 2 or more intronic substitutions were present in 19 of the subjects with intronic SNPs (Additional file 1: Table S2).

In the remaining 675 cases (435 males and 240 females), mutations were not identified in the coding region of the gene, nor in close intronic fragments (around 50 bp in each direction).

The positive predictive value for the enzymatic test in males was 80.43% (CI_95%_: 67.88–92.99%); the method accuracy was 98.12% (CI_95%_:96.80–99.44%) and the false positive rate was 1.88%. These values were calculated taking into account the 37 male patients with confirmed FD diagnosis, among the 479 male subjects that were screened. Mean activity in the whole male cohort was of 7.6 μmol/Lh ±4.02 and median was 3.08 ± 1.57.

The genetic study was not generally performed in males with activity above the cut-off value, however 22 samples from men with α-Gal A activity between 2.6 and 5 μmol/Lh were sequenced by specific request of the responsible physicians, due to their clinical manifestations. Among these patients we identified the 4 subjects with the p.Asp313Tyr polymorphism, the individual with the controverted late onset variant p.Ala143Thr and the subject with a complex intronic haplotype in intronic region (rs2071225, rs5903184, rs2071397 and rs2071228).

### Complex haplotypes analysis

Due to the clinical features presented by 3 of the subjects (#114; #115 and #116), who were found to bear a complex association of intronic variants in *GLA*, we analyzed transcript levels and Gb3 deposits presence in leukocytes samples and skin biopsy, respectively, in order to detect possible defects in gene expression.

Individuals #115 and #116 were a 7-year-old girl and her 42-year-old father, who presented haplotype rs2071225, rs5903184, rs2071397 and rs2071228.

The father referred hearing loss and acroparesthesia associated to microalbuminuria and renal dysfunction. The girl referred erratic pain at the extremities, without pain crisis, recurrent abdominal pain related to food ingestion and poor sweating. Angiokeratomas, skin lesions or organomegaly were not detected. Cardiorespiratory and neurological examinations were normal, as well as renal function.

**Fig. 1:**
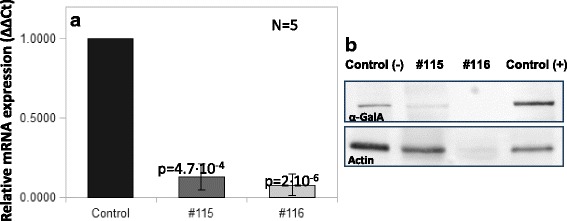
*GLA* mRNA levels (**a**) and Western blot of α-Gal A and actin (**b**) measured in leukocyte extracts of samples from subjects #115 and #116. In panel A, bars represent relative levels of mRNA expression (ΔΔCts) normalized to the control. Two different controls (healthy volunteers’ samples) were used in independent experiments (control bar). In panel B, Control (−) represents the sample from a healthy volunteer and Control (+) represents the sample from a patient diagnosed with FD carrying a missense pathogenic mutation

*GLA* transcription was significantly impaired in these subjects at mRNA level (mRNA 13% of control in #115 and 7% of control in #116), (Fig. 1a), as well as at protein level (α-Gal A expression is decreased compared to actin endogenous control in the Western blot), (Fig. 1b).

**Fig. 2:**
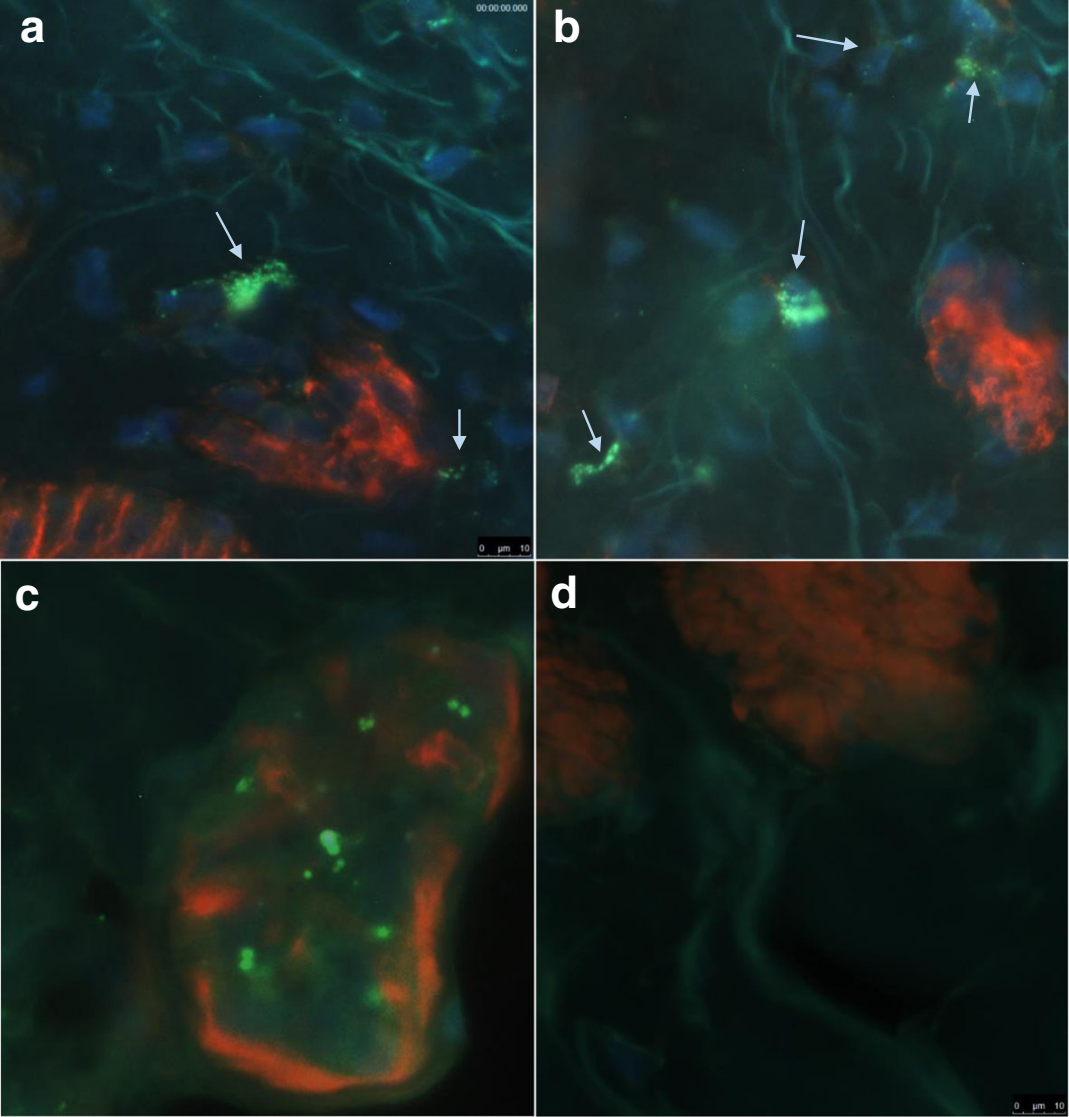
Immunofluorescence of Gb3 deposit in skin biopsy from subject #115. Deposits are stained in green (CD77_FITC) and affected fibroblasts are indicated with arrows (**a**, **b**), blue staining represents nuclei (DAPI) and red staining polymerized actin (Rhodamine-Phalloidin). Panel **c** shows an eccrine gland with green deposits and panel **d** shows a non-affected field of the same biopsy. Collagen fibers are easily detected in all panels due to auto-fluorescence of the specimen

A low percentage of Gb3 deposits (7 affected fibroblasts per examined section) was detected by CD77 immunolabeling in dermis fibroblasts of #115 skin biopsy (Fig. 2a, B). Positive Gb3 cells were also found in one eccrine epithelial gland (Fig. 2c). We were not able to detect the presence of Gb3 deposits in endothelial cells of blood vessels or nerve fibers. Biopsy of patient #116 was not available.

Although these results show that *GLA* transcription is impaired in patients #115 and #116, we cannot confirm the diagnosis of FD in these subjects, since we did not measure Gb3 or Lyso-Gb3 levels in urine or plasma, neither we showed the presence of lamellar bodies.

Patient #114 was a 47-year-old woman, presenting the association of intronic variants rs2071225 and rs2071228 and α-Gal A activity of 4.6 μmol/Lh. She reported multiple clinical symptoms related to classical FD including acroparesthesia, neuropathic pain, hypohidrosis, skin lesions and muscle weakness.

**Fig. 3:**
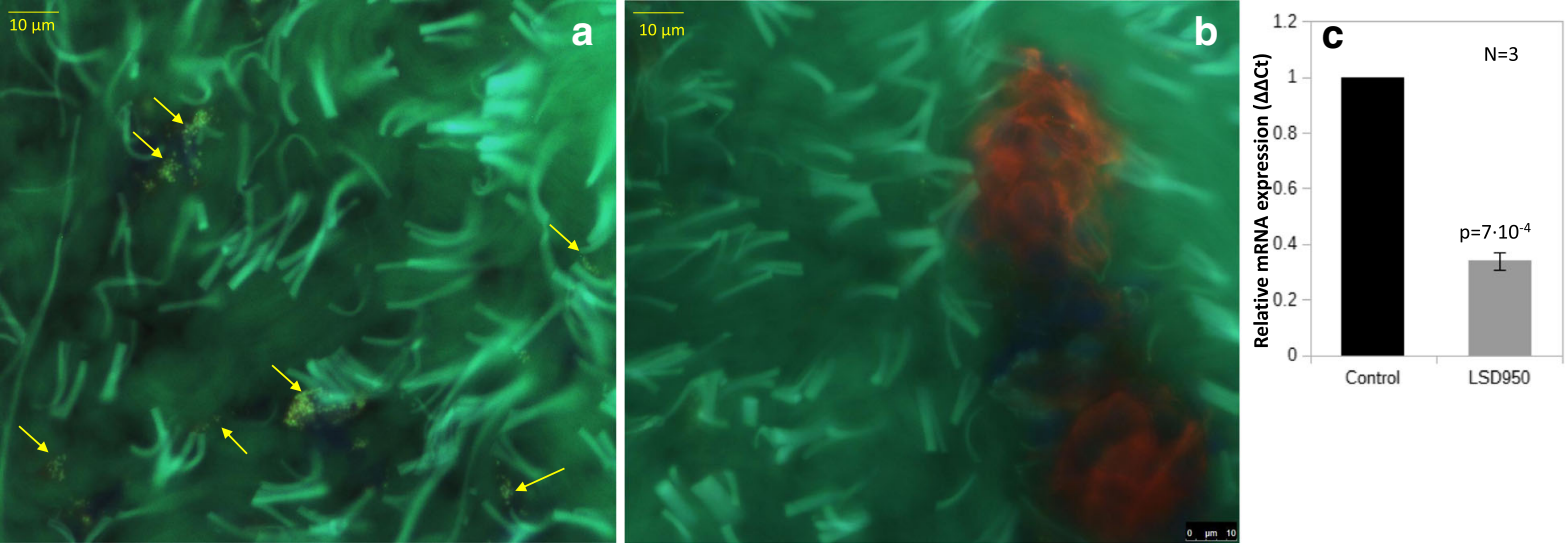
Immunofluorescence of Gb3 deposits (CD77_FITC_green, affected fibroblasts indicated with yellow arrows) in skin biopsy from patient #114 (**a**: affected field; **b**: non-affected field) and *GLA* mRNA levels in leukocyte extract form patient #114 (**c**) compared to sample from 2 healthy volunteers (control bar)

Similarly to the previously analyzed subjects, this woman presented impaired *GLA* transcription (30% of control) and Gb3 deposits in skin fibroblasts (10 affected cells for each examined section) (Fig. 3) were identified through immunohistochemistry. Also in this case Gb3 or Lyso-Gb3 levels in urine or plasma are not available to confirm FD diagnosis.

## Discussion

In this paper, we have shown how a large number of patients with FD can be identified through screening of risk populations (2 or more symptoms related to FD or family history), which stresses that the rate of delayed diagnosis in FD remains excessively high. Late diagnosis is detrimental in FD, since it prevents patients from being treated promptly with the available specific therapies.

Van der Tol et al. [25] conducted a systematic review of FD diagnosis studies including screening of newborns and risk population, to recalculate the prevalence of the disease. According to this article the estimated prevalence of classical FD in high risk populations (45 studies computed) is 0.12%, although this value increases to 0.62% when including all the detected variants in *GLA* (variants of uncertain significance or neutral variants), while it reported a prevalence of *GLA* variants of 0.04% in newborn screenings (6 studies computed). The most recent newborn screenings performed in Washington [10] and Missouri [11] reported an FD prevalence of 0.012% in live births or 0.034% respectively.

The percentage of patients with classic FD in the present study was of 7.72% in the male cohort and 9.56% in the whole group of subjects; however, these values are not representative of the general population, since the sample size is relatively small and subjects selection criteria included individuals with high clinical suspicion (2 or more symptoms), as well as genetically-linked relatives of FD patients, who comprise obligate carriers of mutant alleles.

Moreover, male subjects’ samples were preferentially sent for analysis to our center, by their responsible physicians, since the FD phenotype is usually more easily recognized. The lack of homogeneous selection criteria in our cohort, justifies the extremely high percentage of positive cases, which therefore cannot be directly compared with the prevalence value in risk homogeneous populations estimated by Van der Toll et al. in their meta-analysis of the literature. Our group recently published the results of a screening performed in the Spanish newborns [26], which determine that the prevalence of the disease is 0.013% in males from the Northwest of Spain. Therefore, we estimate that the real prevalence of FD in our country could be around 0.013% or slightly higher considering that the majority of the definitive diagnosis of Spanish adult patients described in the present study was detected in the Mediterranean area, while the newborn screening only includes subjects from the Northwest- Atlantic region.

Although this study pursued reaching a definitive diagnosis in the analyzed samples, due to the lack of homogeneous selection criteria, it was designed as an observational study, which can help to define precise parameters for a validated diagnostic study. The cut-off point that we defined setting up the present screening was of 2.6 μmol/Lh, estimated measuring activity in DBS from 5 FD patients with definitive diagnosis and 15 healthy volunteers. The low number of positive patients available when setting up the assay may have caused an overestimation of the cut-off point, which consequently led to a high false positive rate and a low positive predictive value. If we recalculate the threshold for α-Gal A activity based on the data obtained in the male cohort analyzed in the present work, we will obtain a value of 2.14 μmol/Lh, (percentile of positive patients + 0.5%). If we would have applied this cut-off to our cohort the positive predicted value of the assay would have been 90.24% (CI 79.94–100%) the false positive rate 0.84% and method accuracy 99.16% (CI 98.25–100%),(or Method accuracy 99.3%, positive predictive value 92.68% and false positive rate 0.63%, assuming that p.His125Leu is confirmed to be pathogenic.)

**Fig. 4:**
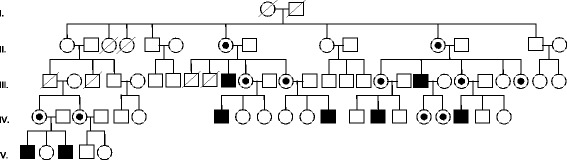
Genetic tree of a family with members affected by FD, who carry the p.Pro205Ser mutation. Circles indicate females, squares indicate males. Black squares are the homozygous male members of the family and black/white concentric circles indicate the female heterozygous patients

The largest family identified in this study, counted with 19 members (11 females and 6 males) with the p.Pro205Ser mutation (Fig. 4). The index case was a male patient with severe hypertrophic myocardiopathy who was diagnosed after 25 years of dialysis and two renal transplants. This is a perfect example of the dramatic consequences of delayed diagnosis for patients and families, which also determines important economic repercussions to the Health Care System.

Nevertheless, without implementing a systematic screening for FD in certain risk conditions (i.e. renal failure, stroke in the young, etc), the non-specificity of the symptoms makes difficult to establish a definitive diagnosis. Indeed, in the large family mentioned, the clinical features were different in each member. Although most of them are affected by renal and cardiac symptoms, two males presented neurological symptoms rather than renal dysfunction and one of them prematurely died due to stroke. None of these patients presented *cornea verticillata*, which is traditionally considered as a hallmark of FD.

The present study also contributed to the identification of five new mutations, which had not been previously described (c.431delG; c.1182delA; c.932 T > C; c.125 T > A, c.778G > A). Pathogenic character of these mutations was predicted by in silico analysis and definitive FD diagnosis was confirmed for these patients, as detailed in the results section, by α-Gal A activity measured in leukocytes (for males only), LysoGb3/Gb3 levels in plasma or urine or biopsy examination.

Moreover, we detected a family bringing the possible pathogenic mutation c.374A > T, p.His 125Leu, which need to be further studied. The index case of this family is a male subject with a α-Gal A activity measured in DBS of ±0.13 0.01 μmol/hL, who presents with clinical symptoms compatible with FD, however we were not able to collect data to confirm a definitive diagnosis of these subjects (ej.Gb3 levels or biopsy).

On the other hand, we also detected 8 individuals with possible late-onset FD, as well as 36 subjects with SNPs in intronic regions (often forming complex haplotypes). These kinds of variants are generally non-pathogenic polymorphisms and their presence is not sufficient to determine FD phenotype [27], for this reason they were not included in the method accuracy calculation. Nonetheless, frequent variants such as p.Arg118Cys (0.04%) and p.Ala143Thr (0.09%) have been associated to late onset FD [4, 13, 25] and the deficient expression of α-Gal A has been reported in subjects with complex intronic haplotypes [28, 29].

As stated by Schiffmann et al. [6], the clinical heterogeneity of FD, even in patients carrying the same mutation, is an evidence that the individual risk of developing complications may depend on α-Gal A deficiency interplay with other factors (i.e. genetic, epigenetic or environmental). An accurate follow-up of subjects presenting variants of unknown significance and a biopsy examination are necessary tools to determine if FD will be eventually developed.

In the present study, we analyzed *GLA* transcription and Gb3 expression in three subjects presenting intronic SNPs, since their clinical features were highly suggestive of FD. Subjects #115 and #116 presented a complex haplotype, previously associated with FD [30–32]. Gervas et al. [32] described *GLA* transcription impairment in a Spanish family with this complex haplotype. The affected members of the family presented galactosphingolipid accumulation in fibroblasts and, in some subjects, glycolipid storage was also present in renal tubules and glomeruli. As addressed in this article, the transcription defect could be the consequence of the alteration of a nuclear protein binding site, caused by SNP rs2071225. Schelleckes et al. [30] also reported FD-related cryptogenic stroke and small fibre neuropathy in two families with intronic SNPs in the *GLA* promoter region and showed that gene transcription is impaired due to inefficient binding of the transcription factor EB to the promoter. Subjects #115 and #116 came from the same geographic area of the family described by Gervas et al. and we cannot rule out that they belong to the same family. Our results are comparable to those described in this article [32] in terms of *GLA* mRNA levels, although the number of affected fibroblasts is low in the biopsy we examined. Since this biopsy was obtained from a 7-year-old girl, the low amount of Gb3 in this specimen may reflect an early stage of an FD progressive condition.

Due to the identified functional and quantitative deficiency of α-Gal A associated to incipient formation of Gb3 deposit in skin biopsy, we conclude that it is possible that subjects #115-and #116 are, in fact, FD patients; however, for a definitive diagnosis, a biopsy examination is required for subject #116 and a second biopsy examination is necessary to confirm storage progression in subject # 115.

We also analyzed the case of subject #114 who is heterozygous for the rs2071225 and rs2071228 SNPs, but shows many clinical signs usually present in classic FD patients. Similarly to #115 and #116, this subject presents a partial impairment of *GLA* levels and functionality, as well as a low detectable amount of Gb3 deposits detected in the skin biopsy. As commented previously, this data are not sufficient to establish definitive diagnosis, although an accurate follow-up of the subject is advisable. Indeed the presence of typical clinical manifestations suggests that α-GalA activity in functional organs such as kidney and heart could be significantly impaired, in spite of the residual activity of 4.66 ± 0.67 μmol/Lh measured in DBS. As mentioned in reference [6], a heart-kidney-cerebrovascular system gradient seems to have been established for FD and a true threshold for pathogenic enzyme activity in tissues is difficult to estimate. Moreover external factors, such as random X-chromosome inactivation, hormone regulated transcription mechanisms and other epigenetic factors, can contribute to develop the phenotype. For all these reasons, a biopsy examination is strongly recommended for specific diagnosis of subject #114, ideally performed in kidney or heart. Skin biopsy is a valuable tool for diagnosis of FD patients, since it is a barely invasive method which allows monitoring of glycosphingolipid storage localization and amount in different structures (nerves, glands, blood vessels) [2]; however a minor presence of deposits in the skin does not exclude a more severe functional impairment in other tissues.

While several recent works point to a non pathogenic role of variants such as p.Ala143Thr and p.Arg118Cys [33, 34], our results as well as other reports of the literature suggest that complex haplotypes of intronic variants may predispose to Gb3 accumulation in tissues, and therefore to the development of FD phenotype in certain subjects, presumably when additional genetic or epigenetic gene modifiers are present. Indeed, also the variant p.Ala143Thr has been occasionally associated to the development of classic FD [34]. Further investigation is required to validate this hypothesis and elucidate the mechanisms involved in *GLA* expression impairment in presence of specific frequent polymorphisms.

## Conclusions

This observational study performed in subjects with high risk to present FD (two or more clinical manifestations or family history of the disease) helped to identify 77 new confirmed patients and 2 individuals with a possible diagnosis of FD, carrying the newly described variant p.His125Leu (among a total of 805 analyzed samples). Additionally, we detected 8 subjects with variants related to possible late onset FD and 3 subjects with SNPs in intronic region associated to functional and quantitative deficiency of α-Gal A. These results confirm that FD is still under-diagnosed in the population and that the real prevalence of the disease is more likely reflected by the values estimated in recently performed newborn screening studies. The wide variety of clinical symptoms and signs showed by each patient, as well as the identification of impaired enzyme functionality and pathological signs in subjects with SNPs in *GLA*, also suggest that additional factors (i.e. genetic or epigenetic) may contribute to determine FD clinical phenotype.

## Additional files

Additional file 1: **Table S1.** List of the primers used for genetic sequencing of *GLA*. Primers align to intronic sequencing flanking exons regions. **Table S2.** Subjects with SNPs in intronic regions of *GLA* detected through genetic study. Enzymatic activity is expressed in μmol/Lh ± SD. NM = not measured; X = not indicated. Bold letters indicate the subjects whose *GLA* transcription levels and biopsy were analyzed. **Figure S1.** Distribution of collected samples. In panel A are represented the relative percentages of medical departments that collected the samples. In panel B it is represented the number of samples collected in each Spanish region. **Figure S2.** An example of blood card used to collect samples, showing collecting instructions and symptoms list with check boxes. **Figure S3.** De novo mutation c.1182delA in *GLA,* Sequencing analysis of the sample from patient # 69: the chromatogram is compared with a wild type control sequence, mutation site is indicated by an arrow (A). Panel B shows predicted changes in the amino acid sequence determined by the nucleotide deletion. Dots at the end of the wild type sequence indicates that the nucleotide sequence continues with additional 24 triplets before stop codons. (DOCX 4204 kb)

## Abbreviations

DBS: Dried Blood Spots
FD: Fabry Disease
Gb3: Globotriaosylceramide
LSDs: Lysosomal Storage Disorders
lyso-Gb3: Globotriaosylsphingosine
SD: Standard deviation
SNPs: Single Nucleotide Polymorphisms
α-Gal A: α-Galactosidase A
