# Supplementary material for: Fabry disease in the Spanish population: observational study with detection of 77 patients
Source: Orphanet J Rare Dis. 2018 Apr 10;13:52. doi: 10.1186/s13023-018-0792-8 (PMC5891901; doi:10.1186/s13023-018-0792-8)
Supplement: Supplementary file 1 — Table S1. List of the primers used for genetic sequencing of GLA. Primers align to intronic sequencing flanking exons regions. Table S2. Subjects with SNPs in intronic regions of GLA detected through genetic study. Enzymatic activity is expressed in μmol/Lh ± SD. NM = not measured; X = not indicated. Bold letters indicate the subjects whose GLA transcription levels and biopsy were analyzed. Figure S1. Distribution of collected samples. In panel A are represented the relative percentages of medical departments that collected the samples. In panel B it is represented the number of samples collected in each Spanish region. Figure S2. An example of blood card used to collect samples, showing collecting instructions and symptoms list with check boxes. Figure S3. De novo mutation c.1182delA in GLA, Sequencing analysis of the sample from patient # 69: the chromatogram is compared with a wild type control sequence, mutation site is indicated by an arrow (A). Panel B shows predicted changes in the amino acid sequence determined by the nucleotide deletion. Dots at the end of the wild type sequence indicates that the nucleotide sequence continues with additional 24 triplets before stop codons. (DOCX 4204 kb) [file 13023_2018_792_MOESM1_ESM.docx]

**Additional files**

**Table S1** List of the primers used for genetic sequencing of *GLA*. Primers align to intronic sequencing flanking exons regions.

| ***Exons*** | ***Sequence*** |
| --- | --- |
| **1f** | 5’ gcccctgaggttaatcttaa 3’ |
| **1r** | 5’ aactgttcccgttgagactc 3’ |
| **2f** | 5’ ggaggtacctaagtgttcat 3’ |
| **2r** | 5’ agcttctgtacagaagtgct 3’ |
| **3f** | 5’CAGTTCTGGGCTATAGCTCACATGC 3’ |
| **3r** | 5’ ttctttggctcagctaccat 3’ |
| **4f** | 5’ agactgaaccccatctcaaa 3’ |
| **4r** | 5’ ccttggtttcctttgttgtc 3’ |
| **5f** | 5’ ctgtaaactcaagagaaggc 3’ |
| **5r** | 5’ caggaactttacctgtatttacc 3’ |
| **6f** | 5’ ggtttctccatatgggtcat 3’ |
| **6r** | 5’ ttaggcccaagacaaagttg 3’ |
| **7f** | 5' tgaatgccaaactaacaggg 3’ |
| **7r** | 5' atgagccacctagccttga 3’ |

**Table S2** Subjects with SNPs in intronic regions of *GLA* detected through genetic study. Enzymatic activity is expressed in μmol/Lh ±SD. NM=not measured; X=not indicated. Bold letters indicate the subjects whose *GLA* transcription levels and biopsy were analyzed.

| *Patient* | *Age* | *Sex* | *α-Gal A activity* | *SNPs* | *Medical Department* |
| --- | --- | --- | --- | --- | --- |
| 95 | 14 | F | 3.16±0.20 | c.-10C>T (rs2071225) | Neurology |
| 96 | 75 | F | 8.03±0.90 | c.-10C>T (rs2071225) | Nephrology |
| 97 | 19 | F | 11.41±3.49 | c.-10C>T (rs2071225) | Dermatology |
| 98 | 66 | F | 9.49±1.38 | c.-10C>T (rs2071225) | Nephrology |
| 99 | 21 | F | 2.12±0.20 | c.-12G>A (rs3027585) | Nephrology |
| 100 | 52 | F | 8.17±0.48 | c.-12G>A (rs3027585) | Nephrology |
| 101 | X | F | 8.39±0.63 | c.-12G>A (rs3027585) | Cardiology |
| 102 | 38 | F | 5.33±0.47 | c.-12G>A (rs3027585) | Laboratory |
| 103 | X | F | NM | c.-30G>A (rs3027584) | Genetics |
| 104 | 45 | F | 2.2±0.09 | c.640-16A>G (rs2071397) | Genetics |
| 105 | 39 | F | 2.94±0.16 | c.370-81_370-77delCAGCC (rs5903184) | Cardiology |
| 106 | 80 | F | 4.71±0.06 | c.370-81_370-77delCAGCC (rs5903184) | Nephrology |
| 107 | 45 | F | 6.08±1.23 | c.370-81_370-77delCAGCC (rs5903184) | Cardiology |
| 108 | 49 | F | NM | c.370-81_370-77delCAGCC (rs5903184) | Genetics |
| 109 | 35 | F | 9.74±3.37 | c.370-81_370-77delCAGCC (rs5903184) | Nephrology |
| 110 | 39 | F | 9.55±408 | c.1000-22C>T (rs2071228) | Dermatology |
| 111 | 50 | F | 5.02±0.48 | c.-12G>A / c.1000-22C>T | Nephrology |
| 112 | 48 | F | 13.62±2.01 | c.-12G>A / c.1000-22C>T | Cardiology |
| 113 | 37 | F | 4.68±1.08 | c.-12G>A / c.1000-22C>T | Laboratory |
| **114** | **X** | **F** | **4.66±0.67** | **c.-12G>A / c.1000-22C>T** | **Nephrology** |
| **115** | **7** | **F** | **4.76±0.91** | **c.-10C>T, c.370-81_370-77delCAGCC, c.640-16A>G / c.1000-22C>T** | **Neurology** |
| **116** | **41** | **M** | **2.71±0.17** | **c.-10C>T, c.370-81_370-77delCAGCC, c.640-16A>G / c.1000-22C>T** | **Neurology** |
| 117 | 55 | F | 11.52±1.37 | c.640-16A>G / c.1000-22C>T | Cardiology |
| 118 | X | F | 6.07±0.72 | c.370-81_370-77delCAGCC / c.1000-22C>T | Internal Medicine |
| 119 | X | F | NM | c.370-81_370-77delCAGCC / c.640-16A>G / c.1000-22C>T | Genetics |
| 120 | 57 | F | 5.55±0.58 | c.370-81_370-77delCAGCC / c.640-16A>G / c.1000-22C>T | Neurology |
| 121 | 59 | F | 8.11±2.91 | c.370-81_370-77delCAGCC (HT) / c.640-16A>G (HT) / c.1000-22C>T (HT) | Genetics |
| 122 | 26 | F | NM | c.-12G>A (HT) / c.370-81_370-77delCAGCC (HT) / c.640-16A>G (HT) / c.1000-22C>T (HT) | Nephrology |
| 123 | X | F | 7,06±0.1 | c.-12G>A (HT) / c.370-81_370-77delCAGCC (HT) / c.640-16A>G (HT) / c.1000-22C>T (HT) | Nephrology |
| 124 | 48 | F | 6.7±2.32 | c.-12G>A (HT) / c.370-81_370-77delCAGCC (HT) / c.640-16A>G (HT) / c.1000-22C>T (HT) | Nephrology |
| 125 | X | F | NM | c.-12G>A (HT) / c.370-81_370-77delCAGCC (HT) / c.640-16A>G (HT) / c.1000-22C>T (HT) | Genetics |
| 126 | 54 | F | 5.46±0.20 | c.-10C>T/ c.370-81_370-77delCAGCC / c.640-16A>G / c.1000-22C>T | Nephrology |
| 127 | 27 | F | NM | c.-10C>T/ c.370-81_370-77delCAGCC / c.640-16A>G / c.1000-22C>T | Dermatology |
| 128 | 65 | F | NM | c.-10C>T/ c.370-81_370-77delCAGCC / c.640-16A>G / c.1000-22C>T | Nephrology |
| 129 | X | F | NM | c.-10C>T/ c.370-81_370-77delCAGCC / c.640-16A>G / c.1000-22C>T | Genetics |
| 130 | X | F | NM | c.-10C>T/ c.370-81_370-77delCAGCC / c.640-16A>G / c.1000-22C>T | Genetics |


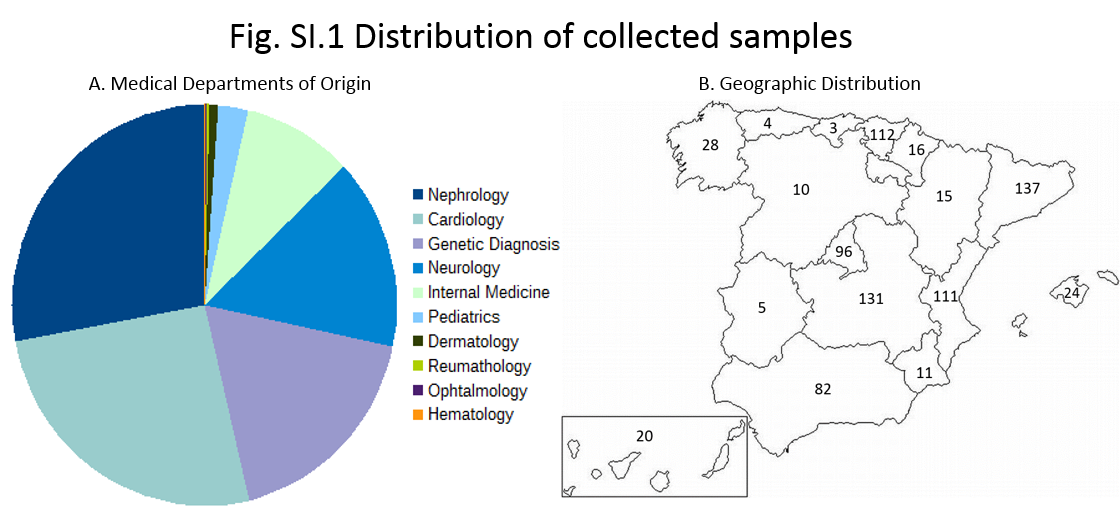


**Figure S1** Distribution of collected samples. In panel A are represented the relative percentages of medical departments that collected the samples. In panel B it is represented the number of samples collected in each Spanish region.

**
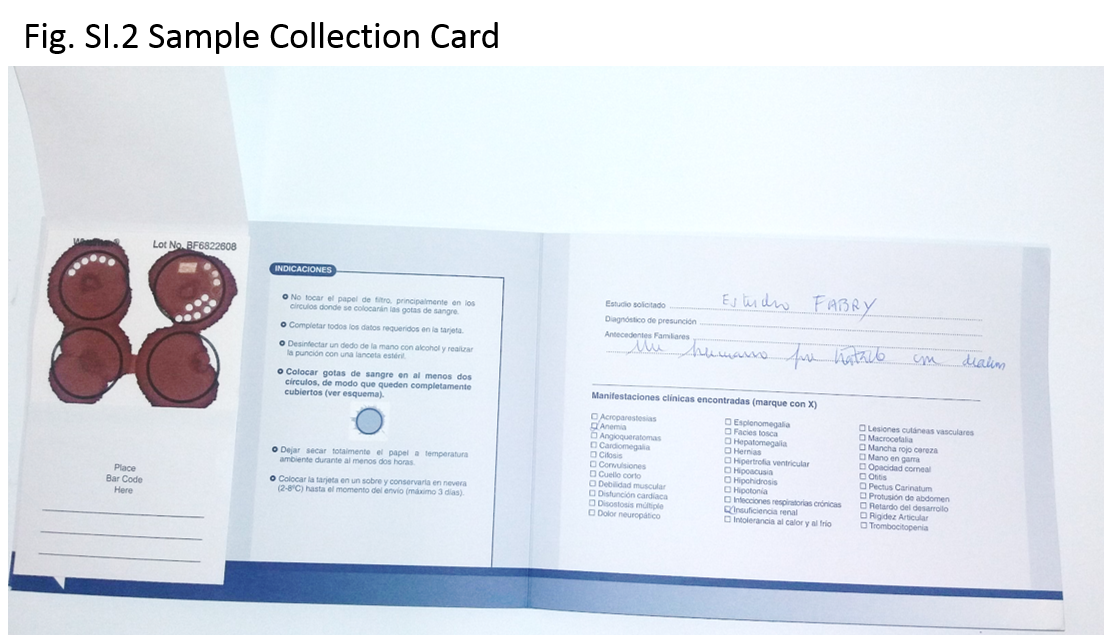
**

**Figure S2** An example of blood card used to collect samples, showing collecting instructions and symptoms list with check boxes.


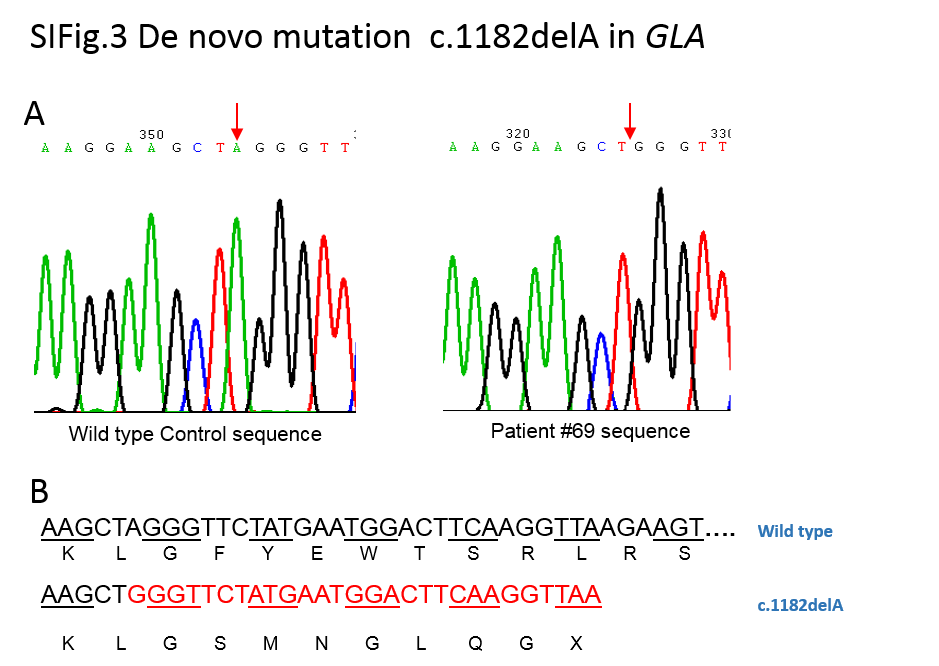


**Figure 3 De novo mutation c.1182delA in *GLA***

Sequencing analysis of the sample from patient # 69: the chromatogram is compared with a wild type control sequence, mutation site is indicated by an arrow (A). Panel B shows predicted changes in the amino acid sequence determined by the nucleotide deletion. Dots at the end of the wild type sequence indicate~~s~~ that the nucleotide sequence continues with additional 24 triplets before stop codons.


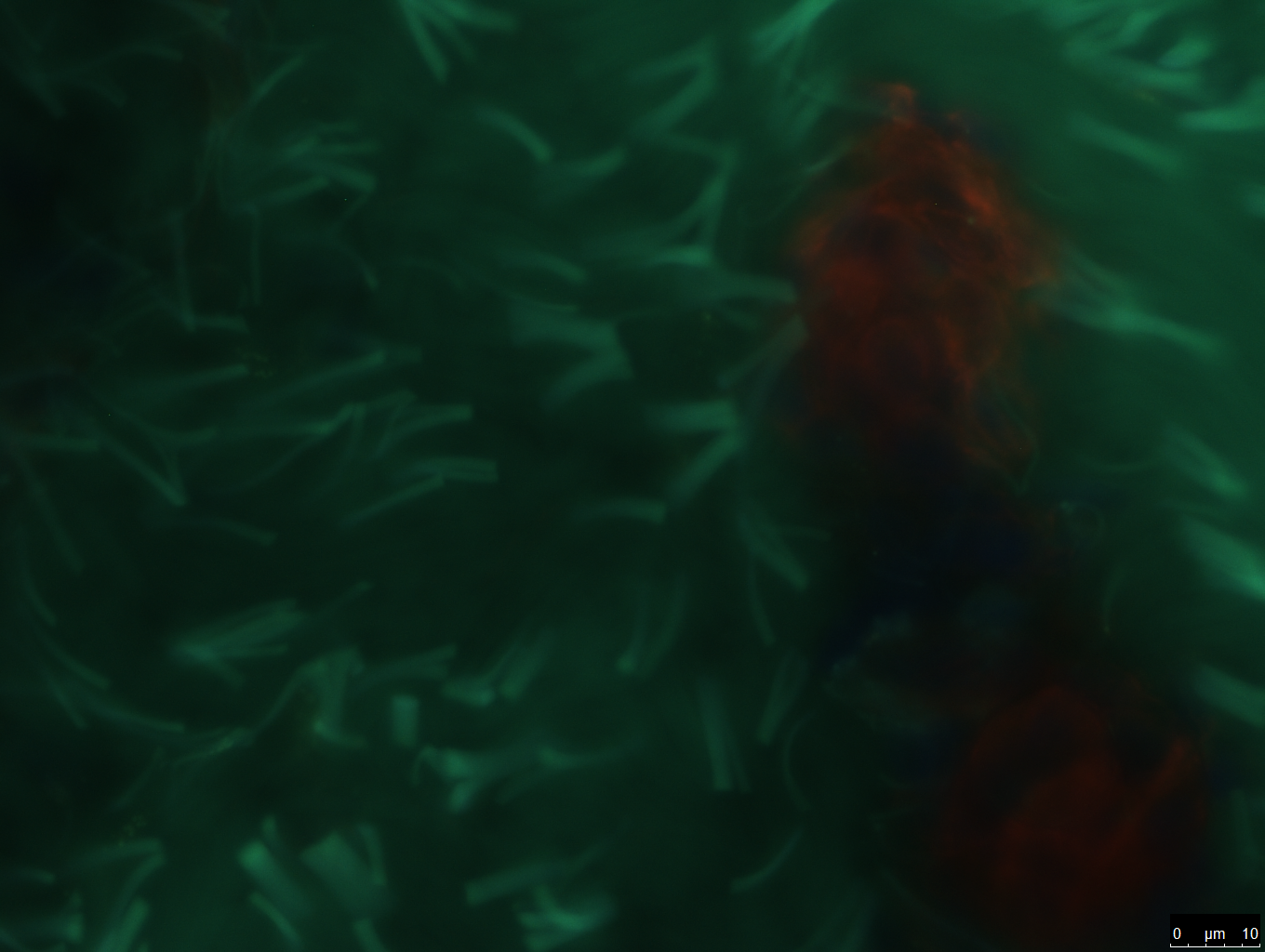


**Figure S4** Immunofluorescence of a skin biopsy from a control subject non affected with FD stained with CD77_FITC; DAPI_blue; Rodamine Phalloidin. No Gb3 deposit is detected. Collagen fibers are easily identified due to auto-fluorescence of the specimen. Sample was obtained from Vigo University Hospital Biobank.
